# Biofilm's Impact on Inflammatory Bowel Diseases

**DOI:** 10.7759/cureus.45510

**Published:** 2023-09-18

**Authors:** Gopal S Palandurkar, Sunil Kumar

**Affiliations:** 1 Department of Medicine, Jawaharlal Nehru Medical College, Datta Meghe Institute of Higher Education and Research, Wardha, IND

**Keywords:** drugs for inflammatory bowel disease, human gut microbiota, epidemiology of inflammatory bowel disease, crohns disease, ulcerative colitis

## Abstract

The colon has a large surface area covered with a thick mucus coating. Colon's biomass consists of about 1,012 colony-forming units per gram of feces and 500-1,000 distinct bacterial species. The term *inflammatory bowel disease* (IBD) indicates the collection of intestinal illnesses in which the digestive system (esophagus, large intestine, mouth, stomach, and small intestine) experiences persistent inflammation. IBD development is influenced by environmental (infections, stress, and nutrition) and genetic factors. The microbes present in gut microbiota help maintain intestinal homeostasis and support immune and epithelial cell growth, differentiation, as well as proliferation. It has been discovered that a variety of variables and microorganisms are crucial for the development of biofilms and mucosal colonization during IBD. An extracellular matrix formed by bacteria supports biofilm production in our digestive system and harms the host's immunological response. Irritable bowel syndrome (IBS) and IBD considerably affect human socioeconomic well-being and the standard of living. IBD is a serious public health issue, affecting millions of people across the globe. The gut microbiome may significantly influence IBS pathogenesis, even though few diagnostic and treatment options are available. As a result, current research focuses more on disrupting biofilm in IBD patients and stresses primarily on drugs that help improve the quality of life for human well-being. We evaluate studies on IBD and bacterial biofilm to add fresh insights into the existing state of knowledge of biofilm formation in IBD, incidence of IBD patients, molecular level of investigations, bacteria that are involved in the formation of biofilm, and present and down the line regimens and probiotics. Planning advanced ways to control and eradicate bacteria in biofilms should be the primary goal to add fresh insights into generating innovative diagnostic and alternative therapy options for IBD.

## Introduction and background

Crohn's disease (CD) and ulcerative colitis (UC) are the predominant subtypes of inflammatory bowel disease (IBD). This may have an impact on the entire gastrointestinal tract (GIT). Chronic IBD is diagnosed by comparing endoscopic, clinical, and histological, along with radiographic data, and comprises a relapsing or remitting course [1,2]. UC is a chronic, recurrent, and idiopathic IBD characterized by significant inflammation and immunological responses (cytokine production and T-helper cell) in the intestinal mucosa affecting adults aged between 30 and 40 years. It has a relapsing and remitting course, and ulcers emerge in the distal large intestine; ultimately, the inflammation spreads over the proximal bowel [3-5]. UC may significantly impact one's standard of living, and if oral medications are unsatisfactory, surgical excision of affected intestines showing ulcer would be needed, resulting in disability. Several microorganisms, including Fusobacterium spp. (species), Shigella spp., adhesive Escherichia coli (AIEC), is being discovered in an inflammatory colon. Until now, no such causative microorganisms have been liable for UC [3-5]. On the other hand, CD exhibits transmural inflammation along with epithelioid granulomas in the GIT tissues with elevated levels of interferon-gamma and T-helper cell (Th1) responses [6,7]. CD primarily affects the lower end of the small intestine, excluding the rectum; skip lesions are typical and primarily impact the young age group. It has a relapsing and remitting nature, and the illness evolves from moderate to severe, causing fistulas and strictures that lead to permanent impairment [6,7]. CD patients typically have higher levels of immunoglobulin G (IgG) antibodies. Bacteroides spp. were the most prevalent bacterial species observed. Compared to 15% in UC patients, it shows total mucous bacteria of about 80%. Additionally, it has been established that AIEC manifests in CD [6,7]. IBD has a complicated etiology induced by several events, with immune dysfunction eventually underlying its progression [8,9]. Biofilms offer several environmental, industrial, and health advantages [10,11]. Bacteria adhere to surfaces and are integrated into the extracellular matrix of proteins, polysaccharides, nutrients, and nucleic acids, to form biofilms [12]. Because endoscopy is necessary to reach these surfaces, studying biofilms in the GIT is far more challenging.

## Review

Methodology

With this review, the following objectives are being pursued.

Biofilm's Impact on IBDs

**Figure 1:**
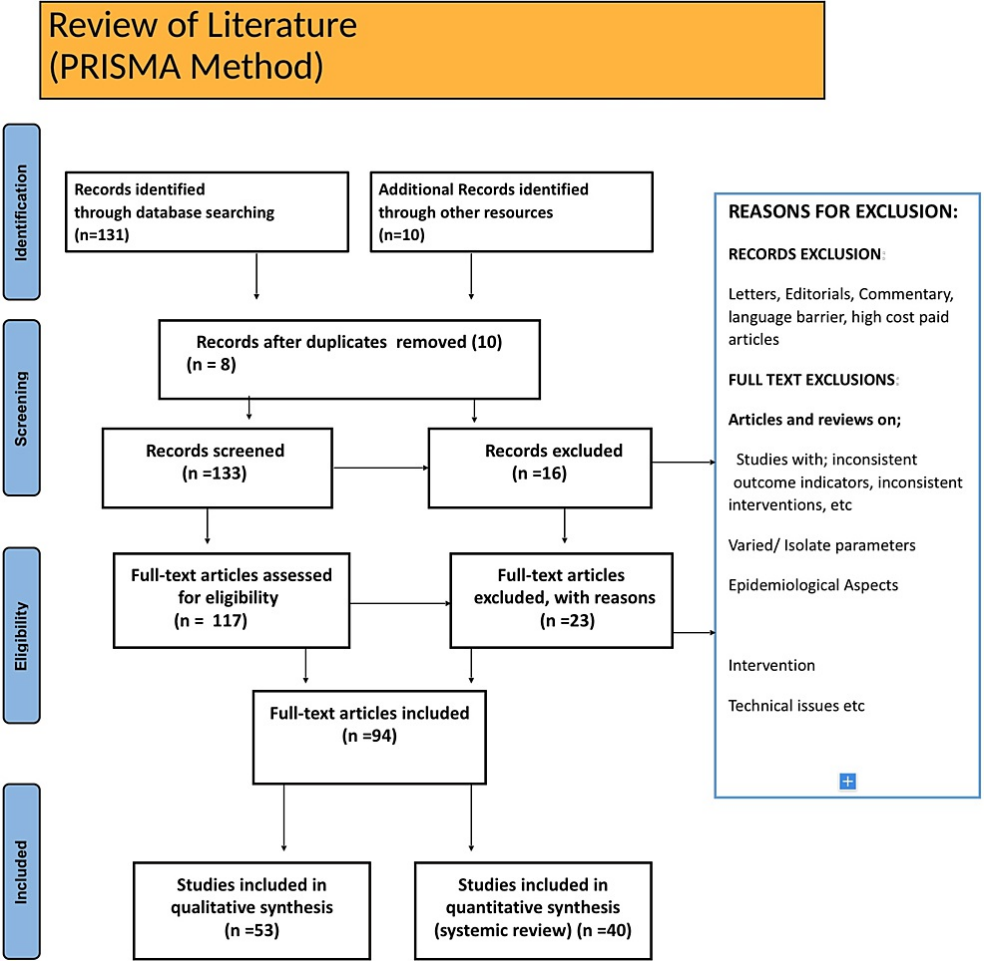
Flow diagram of the literature review. Author's own creation.

Electronic databases such as Google Scholar, Medline, PubMed, and Embase are used to search English-language literature. The search methodology applied a systematic approach to identify relevant studies for the review. The method included searching numerous databases, screening articles, defining the exclusion and inclusion criteria, and selecting the final article for the review. The search terms were "Biofilm," "Incidence of IBD," "Inflammatory bowel diseases," "Techniques for biofilm detection," "ulcerative colitis," "treatment for inflammatory bowel disease," "Crohn's disease," and related synonyms. The writer's experience aided the availability of relevant articles on the topic and expertise. Inclusion criteria encompassed studies devoted entirely to biofilms and bacteria linked to biofilm development in patients with IBD, with a focus on the clinical aspects of IBD diagnosis and treatment options in peer-reviewed articles. Exclusion criteria include nonhuman studies, studies not directly related to IBD, and studies lacking full-text articles and conference abstracts. The search covered only the papers published from the databases' inception until now, with no explicit date constraints. It ensured that the most current study on the subject should be included. Only the articles which are published in English literature are taken into account. The inclusion criteria were satisfied by 94 articles included in the final review. Figure 1 depicts the research methodology showing a flow diagram of the Preferred Reporting Items for Systematic Reviews and Meta-Analyses (PRISMA) approach.

Incidence

Age-specific incidence of patients affected by IBD is more significant in the second and third decades of life, with estimates of over 100-200 per lakh for UC and 6.6 per lakh for CD cases [13,14]. Furthermore, the pervasiveness of IBD in children and adults is rising in developing nations across the globe [15,16]. IBD is a significant public health concern across the globe. It was formerly found in considerable proportions in Australia, western and northern Europe, and North America [17,18]. And yet, it shows a considerable vast geographic distribution and rising frequency in the previously considered low-risk countries. Urbanization, alteration in eating habits (e.g., modernization of Indian food), underlying genetic predisposition, improvements in environmental conditions, and sanitary measures are the major risk factors in the development of IBD [19,20]. Recently, hospital-based studies have revealed a frequent rise in patients with IBD [21]. This might be attributed to increasing physician awareness, better diagnostic processes, and more accessible and improved access to specialized healthcare systems.

IBD risk factors

The prevalence of IBD is significantly impacted by race, urbanization, industry, the hygiene hypothesis, smoking, air pollution, helminthic exposure, and autoimmune disorders. And the body's immune response to these recurring stressors [16]. However, smoking shows a guarding phenomenon in UC, whereas it's an aggravating factor in CD patients. Recent research describes urbanization as a critical risk factor in the development of IBD. Also, air pollution in cities is linked with the development of IBD in earlier stages of life. Occupation exposure, such as manufacturing and driving, has been linked with developing IBD [16]. The hygiene hypothesis states that lack of exposure to helminths or enteric pathogens, better food handling, and hygiene protocol increase the chances of developing IBD in humans [16]. In relatives with UC or CD, there is an 8% to 10% likelihood of developing IBD. Concordance exists between twins with IBD. Genetic factors contribute more to CD than UC in the emergence of IBD. Genetic and environmental factors are needed for the onset of IBD [8]. In newborns born from consanguineous marriage, autosomal recessive mutations in the gene encoding the interleukin-10 (IL-10) cytokine, the IL-10 receptor, and nucleotide-binding oligomerization domain protein 2 (NOD2) have been linked to severe types of CD [8]. It's also common in genetic illnesses that alter glycogen storage disease type 1B and neutrophil function [8].

Molecular mechanism and connection between biofilms and IBDs

Broadly, the formation of a biofilm involves a two-step process comprising adhesion and maturation stages. The adhesion stage initiates with bacteria adhering over the substrate surface and progresses toward the maturation stage, which involves the proliferation and differentiation of related cells. Surface adhesion and cell-to-cell communication signal-controlled pathways were used for both stages [22]. Studies have revealed that bacteria's activity in biofilms boosts adaptive evolution rates under survival-enhancing stress conditions. Many pathways that enhance genomic diversity and selection in survival invent heritable small colony variations (SCVs) [23]. The genetic alterations produced impact various aspects, including bacteria's behavior in a biofilm. Some biofilm-derived variants may have a better dispersal capability, while others may generate biofilm quicker. Besides, an entire of this functionally different microbiota improves biofilm performance to withstand enormous environmental stresses. SCVs have been associated with lifelong infection and in vivo perseverance [24-26]. Studies showed quorum sensing (QS) as a communication mechanism in Pseudomonas aeruginosa strain's biofilm activities [23]. Another experiment showed that the planktonic batch culture showed no variants; later, when the period was extended to five days, it showed SCVs. Several biological processes are affected by diversity, including motility, dietary needs, secreted product synthesis, colony architecture, and three biofilm phenotypes: hyper biofilm development, rapid detachment, and more excellent biofilm-mediated resistance [23]. Gram-negative bacteria release pyomelanin, which increases UV tolerance and host defenses. The hyper-detachment phenotype of the micro variant may provide a benefit to the population by enhancing the dispersion and colonization of new places [23]. Staphylococcus, Pseudomonas, and Enterobacteriaceae colonies were 10 times smaller than usual, and SCVs could revert to average growth [25]. Bacteria that hold onto a surface and form a biofilm are immune to conventional antibiotics. Bacteria in biofilms are enveloped with an exopolysaccharide matrix that might limit antibiotic diffusion [25]. 

Microbes causing biofilm formation

Various microbial species, such as bacteria, fungi, and archaea, reside in the human gut's complex anaerobic environment. Culture-independent 16S rRNA study is employed to investigate the microbial diversity in the gut. According to these studies, the GIT is dominated by Gram-negative Bacteroidetes along with Gram-positive Firmicutes, with methanogens and Actinomycetes occupying minor roles [27]. The vast majority of Firmicutes have been identified as butyrate-producing bacteria or clostridia. Several Actinomycetes and Proteobacteria were also discovered, with Bifidobacteria (an Actinomycetes subtype with health-promoting qualities) accounting for 5% of the microbiota. Methanobrevibacter smithii and Methanosphaera stadtmanae exhibited archaeal diversity. Blastocystis species (unicellular along with several multicellular protists) and scanty fungi concern the Basidiomycetes or Ascomycetes, with the mass belonging to the genus Penicillium glabrum, Candida glabrata, Candida albicans, Penicillium verruculosum, Penicillium sacculum, and Penicillium italicum [27]. Several investigations aimed at differentiating resident microorganisms in healthy persons' guts from those in the guts of patients with IBD discovered a universal decline in bacterial variance in IBD. Furthermore, there was a decline in methanogen variance and an incline in fungal variance in GIT of patients with IBD [28,29]. Microbial communities crowded over inflamed gut surfaces; moreover, the microbial communities in healthy gut tissues did not vary. Several studies have also discovered dangerous microbes in GITs of patients with IBD. C. albicans, Chlamydia pneumonia, Saccharomyces cerevisiae, Mycobacterium avium subspecies paratuberculosis, Listeria monocytogenes, and AIEC are all identified as more potent pathogenic microorganisms in the transmission of CD. Bacteroides, Eubacteria, and Peptostreptococcus count increase in CD, whereas Bifidobacteria levels decrease dramatically [30-32]. 

**Figure 2:**
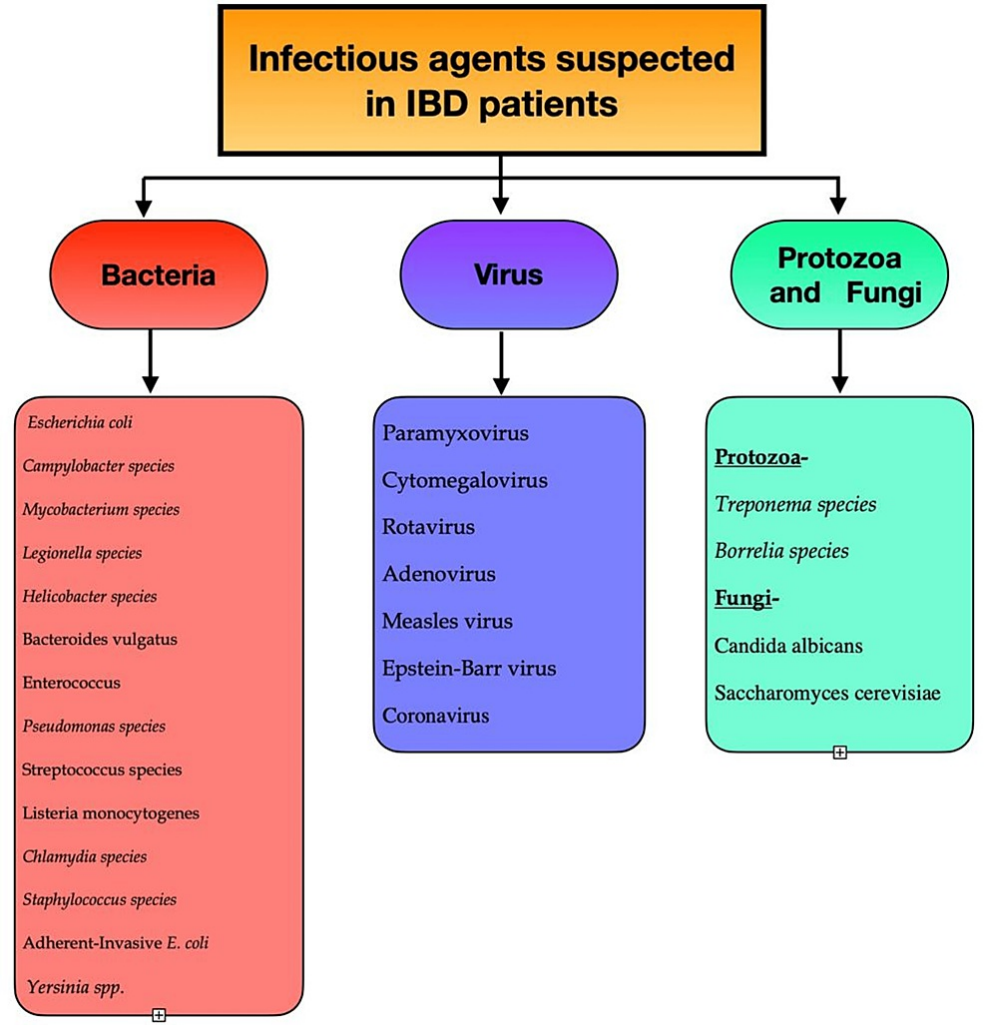
Infectious agents suspected in IBD. Author's own creation. IBD, inflammatory bowel disease

In addition, the frequency of facultative anaerobic bacteria is higher in UC. E. coli is being demonstrated to promote the production of cytokines in inflamed guts of patients with IBD. In genetically susceptible hosts, AIEC, a facultative pathogen, has been related to CD [30-32]. Besides, these studies have found the link between IBD and infections, and the pathogenic bacteria that causes CD or UC is unknown. IBD may be caused by an alteration in the overall microbe population in the GIT (intestinal microbe biofilms) rather than a specific microbial infection [33,34]. It might also be caused by mistaking common, commensal bacteria as pathogens, resulting in inflammation and immune responses. In terms of species along with concentration, a transition in intestinal microbiota or misrecognition by the individual's immune mechanism is defined as genetic dysbiosis [33,34]. These constantly shifting gut microbiota can change the genes' expression in various gastrointestinal functions. Furthermore, it can induce inflammation and sickness in genetically susceptible patients with immune response gene mutations or polymorphisms [33,34]. Figure 2 describes the classification of organisms involved in patients with IBD [35].

Molecular techniques for biofilm detection

Biofilms are highly structured bacterial populations that bind abiotic surfaces such as medical equipment to biotic surfaces like the host cells, and these are surrounded by a self-formed extracellular polymeric matrix [36]. Because of their tenacity, they are resistant to many antimicrobials and escape from the human defense system. Biofilm-forming microbe infections remain substantial clinical problems [37]. The development of biofilms is linked with increased morbidity and mortality rates, as well as hospital expenses, especially devices needed like implants and catheters [38]. Despite years of study, identifying biofilm-related disorders remains challenging since people affected with biofilms do not have distinct symptomatic manifestations and presentations. Even if there are established protocols for recognizing biofilms in research facilities, there is yet to be an equivalent strategy in clinical practice. 

In Vitro Techniques for Biofilm Detection 

Direct observation techniques, such as light microscopy, electron microscopy, and confocal microscopy, can be categorized as imaging techniques that may be used to investigate the complexity and dynamics of biofilms. Researchers might use these technologies to analyze biofilms and visualize their three-dimensional (3D) dynamics [39]. The light microscope is the most convenient and time-efficient approach for investigating the analysis of microorganisms adhering to surfaces and semi-quantitatively assessing the number of microorganisms adhesive over the surface (present, absent, abundant, or unusual) [40]. Following sonication or fluorescence in situ hybridization (FISH), electron microscopy can visualize microbial aggregation. The most widely used appliance is confocal laser scanning microscopy (CLSM), transmission electron microscopy (TEM), and scanning electron microscopy (SEM) [41]. This method's high resolution enables visualization of biofilms visualization, direct detection, and thorough structural findings [41]. In TEM, along with SEM the samples needed to be fixed, then they are dehydrated and later dyed, all of which might alter the form and structure of the biofilm under study [41]. The CLSM has the advantage of presenting a detailed view of the 3D biofilm structure, granting the recognition of macromolecules, the components residing in the biofilm, and the extracellular matrix [41]. In the FISH technique, probes bind over the ribosomal RNA of the spotted organisms, which can be used to recognize specific microorganisms in varied biofilm populace [42]. Because the number of ribosomes in the individual bacteria precisely correlates with its growth action, FISH may be used to estimate the growth rate of organisms in biofilm. The FISH method allows prompt sample testing without any prior preparation and quickly detects microbial aggregation [43]. Although in vitro biofilm research has undoubtedly contributed to our conclusion of biofilm biology, various current methodologies analyze biofilms in conditions other than those in which they are formed [44]. 

These indirect observation techniques can be categorized as follows: tube formation of biofilm, the Congo Red Agar (CRA) test, microtiter plate assay, roller plate method, and polymerase chain reaction. The roll plate technique is a semiquantitative method of analysis, which helps investigate desirable microbial colonization over the outer surface of cylindrical devices like vascular grafts and catheters [45]. In the procedure to count the number of the colony-forming unit, the tip of therapeutic devices is rolled backward and forward onto the agar plate surface and later incubated and counted. Microtiter plate assay is a quantitative method of biofilm detection performed with a microtiter plate reader. It is a low-cost approach for testing several samples simultaneously [46]. Depending on the colony color variations on the CRA test. CRA identifies biofilm-producing microorganisms. It's a brief, sensitive, yet qualitative examination. On CRA, the black colonies indicate more potent biofilm creation, whereas a lack of biofilm production is identified by detecting red colonies [47]. Tube biofilm formation (TBF) test is a technique for detecting the presence of bacteria that produce biofilm. Isolates are placed in plastic tubes and cultured for over 24 hours at 37 °C. Nonadherent bacteria are washed away with buffered solutions, whereas sessile isolates that form biofilms over the sides of polystyrene test tubes are inked with crystal violet or safranin. After air-drying, discolored films on the bottom and sides of the test tubes indicate biofilm production [48-50]. Polymerase chain reactions (PCRs) detect biofilm-associated genes and diagnose specific infections by amplifying specific nucleic acid sequences, even in uncultured clinical samples after sonication [51]. For identifying biofilm-forming microorganisms in clinical samples such as blood, urine, cerebrospinal fluid, wound samples, pleural fluid, and sputum, reverse transcriptase PCR, multiplex PCR, and real-time PCR are all used [52]. 

In Vivo Biofilm Detection Methods

Due to the ethical and logistical challenges, participating in such experiments requires studies that utilize models of mammalian origin for the in vivo biology of biofilm. Optical coherence tomography (OCT) and low-coherence interferometry (LCI) and QS are the types of techniques used for in vivo detection of biofilms. Both OCT and LCI are noninvasive approaches and use a higher resolution of deeper range and imaging [53,54]. Despite breakthroughs in research over the last 30 years, microorganisms that form biofilms remain a severe public medical issue because biofilms are associated with an elevated risk of antibiotic resistance and a worse patient outcome prognosis [55]. Since current detection tools are considerably efficient in research settings and clinical approaches, biofilms and their clinical recognition remain unsolved. Furthermore, studies are required to develop more trustworthy and efficient strategies for faster biofilm detection and raise the possibility of more efficient ways of infection control used in clinical practice [55]. Two potential strategies involved the analysis of essential metabolites present in biofilms of the stool samples and the identification of bacteria that are often found in biofilms. By QS, we can recognize the extracellular signals that bacteria produce and release in the form of chemical substances called *auto-inducers*. Gene expression varies in all bacterial cells when a particular number of auto-inducers are present. This fluctuation in gene expression is linked to differences in cell population density [56]. QS communication in bacteria affects various physiological processes, such as antibiotic production, biofilm formation, motility, competence, symbiosis, sporulation, conjugation, and pathogenicity. Auto-inducers can communicate both within and across bacterial species. Oligopeptides are the autoinducers produced by Gram-positive bacteria and acylated homoserine lactones produced by Gram-negative bacteria [56,57]. Golińska et al. [58] studied enterococci (Enterococcus faecalis) pathogenicity in IBD. Several genes that code for virulence factors are expressed (extracellular surface protein, gelatinase, hyaluronidase, and cytolysin) and are studied in control groups and patients with IBD. Such strains are also linked with QS genes fsrA-C, which regulate by synthesizing such virulence factors [58]. Table 1 summarizes the whole of the molecular methods of biofilm detection [35].

**Table 1:** Techniques of biofilm detection. Author's own creation.

Techniques of biofilm detection	Working principle
In vitro techniques of biofilm detection	Direct observation: electron microscopy, fluorescence in situ hybridization, light microscopy. Indirect observation: roll plate method, microtiter plate assay, Congo Red Agar test, polymerase chain reaction
In vivo techniques of biofilm detection	Low-coherence interferometry, optical coherence tomography, and quorum sensing

Treatment options for IBD

Anti-inflammatory Drugs

5-Aminosalicylic acids (5-ASAs) are anti-inflammatory medicines often utilized in the treatment of UC patients. Moreover, 5-ASA-based medications show little-to-no effect in resolving tissue inflammation and clinical symptoms in CD patients [59,60]. 5-ASAs are more effective medications in the beginning and maintain remission in UC patients, and they have also been associated with a decreased incidence of colitis-related cancers in such individuals [59]. Specific techniques for acting 5-ASAs are being proposed, including cyclooxygenase inhibition, oxygen-free radical, suppression of pro-inflammatory cytokine production, prostaglandin synthesis reductions, mast cell activation, neutrophil chemotaxis blockade, and lipoxygenase inhibition.

*Biological Anti-TNF Agents* 

Certolizumab pegol, adalimumab, golimumab, and infliximab are anti-tumor necrosis factor (anti-TNF) medications frequently used as therapeutic drugs in patients with IBD. Infliximab was more effective for inducing and maintaining remission in UC and CD and therapy of CD fistulas [61]. Previously, infliximab biosimilars of biological products were structural, and adalimumab biosimilars are already under development and are clinically equivalent to a previously Food and Drug Administration (FDA)-approved biological reference product licensed for clinical therapy [62]. In treating IBD, anti-TNF drugs such as infliximab are coupled with immunosuppressive therapies such as azathioprine. Combining azathioprine and infliximab is better than monotherapy with either medication for producing corticosteroid-free clinical remission in UC and CD [63].

Immunosuppressive Drugs

Old immunosuppressive therapies are used for IBD therapy, such as 6-mercaptopurine, methotrexate or azathioprine, and tacrolimus or cyclosporine-A [64-66]. Several medications are proven to establish and maintain remission in clinical studies in persons with UC, and the use of a drug named methotrexate is tested for inducing and maintaining remission in CD. CD and UC patients have commonly shown good outcomes with azathioprine. The latest in vitro and in vivo studies are carried out to study these drugs' possible mechanisms of action. Tacrolimus and cyclosporine-A bind to particular intracellular receptors such as immunophilins and block the nuclear factor of activated T cells (NFAT), a transcription factor affecting lymphocyte apoptosis resistance and cytokine gene transcription [67].

Surging Targets in Tissue Remodeling and Fibrosis

In patients with IBD, matrix metalloproteinase 9 (MMP9) governs tissue remodeling and degradation. MMP9 pathogenic function and expression increase in patients with IBD, particularly UC [68]. MMP9 has been studied for its involvement in inflammation and the function of intestinal barriers. MMP9 is a metalloproteinase associated in IBD with an extracellular matrix degrader and an activated angiogenic switch. The goal of these studies is to estimate MMP9 levels in UD and CD sera in a single experimental setting and assess the potential compared to other biochemical indicators and chosen pro-inflammatory and angiogenic factors; it has much more diagnostic potential [69].

Transcription Factor Inhibitors That Inhibit Cytokine Gene Transcription

Transcription factors that regulate the expression of cytokine genes may be upcoming therapeutic medications in IBD [70,71]. RAR-related orphan receptor-gt (ROR-gt) controls pro-inflammatory T-helper 17 (TH17) cell development and may be a therapeutic target in inflammatory and chronic autoimmune diseases [70,71].

New Blockers of Pro-inflammatory Cytokine Signaling 

Fontolizumab, an anti-interferon-gamma (anti-IFNg) antibody, is being demonstrated to be ineffective in treating active CD [72]. Fontolizumab has a low immunogenicity and is well permissible. Furthermore, due to the therapeutic IL-17A's impact on gut epithelial cells, the anti-IL-17A antibody secukinumab aggravated CD in many individuals [72].

Integrin Blockers Impact T-Cell Trafficking

A potential approach for therapy in intestinal inflammation is inhibiting activated cells [73,74]. In CD clinical studies, Natalizumab, an anti-alpha 4 integrin antibody, was utilized to prevent T-cell homing to the inflamed GIT with a4b7 integrin [75].

Barrier Function Enhancement and Anti-inflammatory Pathway Activation

Gram-negative bacteria named E. coli Nissle (EcN) generate anti-inflammatory proteins and modulate intestinal barrier function [76,77]. EcN is a well-established therapy for UC patients, with anti-inflammatory properties equivalent to 5-ASA [77].

Fecal Microbiota Transplantation

An ill individual consumes healthy donor feces to reestablish healthy microbiota and treat IBD. These have been used extensively in patients with IBD, with mixed-to-moderate results. Long-term benefits require repeated injections. With the prospect of spreading hazardous pathogens in mind, current research suggests using fake stool with minimal risk of infection [78,79].

Alteration in Diet

Patients with IBD should have a well-balanced, high-fiber diet heavy in vegetables, fruits, and grains. Protein-rich diets, alcoholism, and red meat are not recommended in patients with IBD. Administration of these products by patients with IBD may aggravate their disease or relieve inflammation. Specific carbohydrate diets excluding milk, wheat, and sugar have been demonstrated to improve microbial diversity in certain patients with IBD [80,81].

Probiotics

When taken, probiotics are living organisms (certain bacterial strains) that help provide health benefits along with protective and regulatory functions to the body. Probiotic bacteria present in yogurt include Lactobacilli, Bifidobacteria, and Streptococci. Probiotics are more useful in UC patients than in CD patients. EcN 1917 and VSL#3 are the most widely used probiotics in IBD. Such bacteria promote anti-inflammatory bacterial growth while reducing the amount of pathogenic bacteria [82,83].

Case studies conducted in India

Because of neglected healthcare facilities, a lack of trustworthy data collection, and a lack of patient-based investigations, detailed studies on the epidemiology and etiology of IBD are scarce in India [84,85]. Majorly, IBD has been reported in American and European nations in the last two decades, and India has seen a rise in IBD cases. In Table 2, we have included various research that may involve IBD and its specific pathogen [86-94].

**Table 2:** Overview of research conducted in India that involve IBD and its specific pathogen. Author's own creation. CD, Crohn's disease; UC, ulcerative colitis; IBD, inflammatory bowel disease; AIEC, adhesive invasive Escherichia coli; spp., species; PrfA, positive regulatory factor A

Main author of relevant case studies	Outcome
Verma et al. [86]	According to reports, gram-positive Eubacterium and Peptostreptococcus levels increased in CD patients but not in UC patients, although Campylobacter species levels showed a significant rise in both patients. Thus, different sets of bacteria are involved in the pathogenesis of CD and UC.
Banerjee et al. [87]	By analyzing stool samples and rectal biopsies, this study was able to describe the occurrence of viral and parasite infections (such as Strongyloides stercoralis, Ancylostoma duodenale, Entamoeba histolytica, etc.) in UC patients.
Tripathi et al. [88]	It was discovered that 80% of stool samples of UC patients showed positive for Salmonella species.
Bamola et al. [89]	This study proclaimed that patients with colon cancer and patients with IBD had more Bacteroidetes than Firmicutes.
Iyer et al. [90]	They conducted research that explored the relationship between Clostridium diffusively and UC and reported the existence of infection in UC patients leads to deteriorated conditions.
Patra et al. [91]	This study revealed the detection of different serogroups of AIEC in distinct rectal samples and linked it to CD and epithelial injury.
Tirumalai et al. [92]	The authors discussed the food-borne potential pathogen Listeria monocytogenes in India. In addition, the expression of the virulence gene regulator PrfA in L. monocytogenes-infected mammalian hosts was established, which aids in the production of virulence and resistant biofilms.
Sharma et al. [93]	They examined the antimicrobial action of Lactobacillus spp. from curd and milk against several human pathogens such as Staphylococcus aureus, pneumonia, Escherichia coli, Pseudomonas aeruginosa, Listeria monocytogenes, Klebsiella, Bacillus cereus, Shigella flexneri, and Salmonella typhi.
Kaur et al. [94]	Lactobacillus spp. was effective against the diarrhea-causing Vibrio cholerae, which causes substantial mortality in impoverished nations such as India.

## Conclusions

IBD has emerged as a public health concern affecting millions of people. In the last two to three decades, IBD cases in developing countries, including India, have suddenly risen. Certain genetic and environmental risk factors increase the likelihood of developing IBD. The anatomy and physiology of the human colon is highly complex. GIT has a moist and warm environment as it resides in hundreds of bacterial species. In GIT, there are constant environmental and genetic stresses; hence, bacteria become resistant to such stressors and find adaptation as their only method of survival. Hence, the habituation of biofilm affecting the potential of GIT and specific distinct changes continue the progression of IBD. The understanding of gut microbiota in terms of composition and its complex interaction in normal and diseased conditions has been assisted by molecular techniques. Many molecular approaches have aided in understanding gut microbiota composition and complicated interaction in an individual's pathological and normal states. Although several studies (as discussed above) have shown correlations between IBD and infections, the role of a specific pathogenic microbe in UC or CD remains debatable. Although Fusobacterium spp. (species), Shigella spp., and adhesive E. coli are associated with UC patients, whereas Bacteroides spp. are associated with CD patients. It is worth mentioning that significant relief has been shown by integrating probiotics into the daily routine of an IBD patient. Several probiotic strains are beneficial. As a result, probiotics are beneficial for long-term conditions in IBD patients; nevertheless, this paper has limited its discussion to probiotics in IBD patients. Although an extensive study has been conducted on IBD and gut microbiota, the molecular basis of their pathogenicity and biofilm formation remains unknown. It has a delayed impact on developing novel therapeutic drugs for IBD, which would have benefited many of the world's ill population. Thus, more study is needed to understand the pathogenesis of IBD, which could help develop more robust treatment strategies against it. Although several substances (often present in Phyto compounds, foods, oxidizing agents, and synthetic compounds) have been proven to prevent biofilm improvement in the GIT, they are inadequate, and the molecular targets of these compounds remain unknown. Connecting these linkages between the knowledge fields is long overdue, and it will eventually lead to newer methods of eradicating and controlling bacteria in biofilms and newer diagnostic and therapeutic paths for IBD.
